# 周围型肺癌支气管内超声支气管充气征及其病理学基础

**DOI:** 10.3779/j.issn.1009-3419.2010.05.09

**Published:** 2010-05-20

**Authors:** 静 李, 正贤 陈

**Affiliations:** 510080 广州，广东省医学科学院，广东省人民医院呼吸内科 Department of Respiratory Medicine, Guangdong General Hospital, Guangdong Academy of Medical Science, Guangzhou 510080, China

**Keywords:** 支气管内超声, 周围型, 肺, 诊断, Endobronchial ultrasound, Peripheral, Lung, Diagnosis

## Abstract

**背景与目的:**

支气管内超声图像中，良恶性病变均可见支气管充气征。本研究结合病理切片分析支气管内超声图像中不同病变支气管充气征的特征及临床意义。

**方法:**

2005年6月1日-2008年12月30日期间，经胸部X线、CT检查发现肺部周围型病变，经常规可曲支气管镜（以下简称“支气管镜”）检查，明确病变位于段支气管开口以下者92例，采用“径向支气管内超声探头”（radial endobronchial ultrasound probe）进行支气管内超声检查。

**结果:**

78例病灶良恶性诊断明确者纳入分析，恶性病变无支气管充气征者占46.8%（22/47），25例无支气管充气征的病灶中22例为恶性（占88%），其中小细胞肺癌占66.7%（2/3），非小细胞癌占43.9%（18/41），低分化腺癌占50%（5/10），相应病理切片未见支气管充气相。不规则支气管充气征者占51.1%（24/47）。恶性病变中无支气管充气征和不规则支气管充气征两者共计97.9%（46/47），仅1例恶性病变（中分化腺癌），表现为规则的支气管充气征（1.3%）。恶性病变中不规则支气管充气征以腺癌多见，占55.2%（16/29），病理切片见散在支气管充气相，类似征象亦见于2例中分化鳞癌和1例低分化鳞癌。良性病变见规则同心圆状分布支气管充气征者占80.6%（25/31），无支气管充气征者或见不规则支气管充气征各占3.8%（3/31）。

**结论:**

支气管内超声图像于低回声病灶中无支气管充气征或出现不规则支气管充气征时，高度提示恶性病变，出现规则同心圆状分布的支气管充气征时，以良性病变可能性大。

## Abstract (en)

**Background and objective:**

Air bronchogram were visible in endobronchial ultrasound images of benign and malignant lesions. The aim of this study is to analyze the endobronchial ultrasound images of different characteristics of air bronchogram and clinical significance combined with pathologic section.

**Methods:**

From June 1, 2005 to December 30, 2008, 92 patients were found pulmonary peripheral lesions by X-ray and CT examinations, and confirmed that lesions were located lower to the segmental bronchial orifice by flexible bronchoscopy examinations. These patients were examined by radial endobronchial ultrasound probe afterwards.

**Results:**

Seventy-eight patients clarified with benign or malignant diagnosis of lesions were analyzed, among whom, 22 of 47 (46.8%) were of malignant lesion without air bronchogram, 22 patients in 25 of lesions without air bronchogram were confirmed malignant (88%), among whom, 66.7% (2/3), were of small cell lung cancer, 43.9% (18/41) were of non-small cell lung cancer, and 50% (5/10) were of poorly differentiated adenocarcinoma, no sign of air bronchogram was found in corresponding pathological sections. Among those patients with malignant lesion, 51.1% (24/47) were of irregular air bronchogram. For patients with malignant lesions, irregular air bronchogram was most commonly found in adenocarcinoma, 55.2% (16/29) of pathological sections showed signs of air bronchogram, similar sign was also found in 2 patients with moderately differentiated adenocarcinoma and 1 patient with poorly differentiated adenocarcinoma. The total percentage of patients without air bronchogram and with irregular air bronchogram was 97.9% (46/47), only one of them (with medium differentiated adenocarcinoma) showed regular air bronchogram (1.3%). 80.6% (25/31) patients with benign lesion showed regular sign of air bronchogram distributed in the shape of concentric circles, the percentage of patients without air bronchogram or with irregular air bronchogram was both 3.8% (3/31).

**Conclusion:**

When there's no air bronchogram, or irregular air bronchogram sign found by endobronchial ultrasound examination, it is highly indicated that there is a malignant lesion, while there's regular sign of air bronchogram distributed in the shape of concentric circles, it is likely to be a benign lesion.

普通可曲支气管镜检查于直视下往往不能发现肺周围型病灶；经体表超声检查肺周围型病灶仅限于紧贴胸壁的病灶。支气管内超声探头的外径2.0 mm-2.5 mm，远小于普通支气管镜的外径（4.9 mm-6.0 mm）。可以通过支气管镜的活检通道（直径2.0 mm-2.8 mm）^[1]^送入肺周围型无气体或少气体病灶部位，甚至可达胸膜下，直接到达病灶内部进行扫描，减少了气体及其它组织器官的干扰，而正常肺组织所含气体对超声波产生强反射，与病灶之间形成鲜明对比。由于支气管内超声所使用的探头为高频探头，可以非常清晰地显示病灶及其内部的细微结构，如细小的血管、支气管、囊腔等。目前根据支气管内超声图像特征对肺周围型病灶进行良恶性诊断的研究较少。我们既往报道^[2]^支气管内超声图像特征对病灶良恶性的鉴别具有一定诊断价值，其中的一项指标为病灶内的支气管充气征，在超声图像上表现为点线状高回声，本文重点分析该项指标的特征和临床意义。

## 1 材料与方法

### 1.1 病例来源

2005年6月1日-2008年12月30日期间，广东省人民医院支气管镜室连续就诊的门诊和住院患者中，符合入选标准者纳入研究。

### 1.2 入选标准

经胸部X线、CT检查发现肺部周围型病变，经常规可曲支气管镜（以下简称“支气管镜”）检查，明确病变位于段支气管开口以下者。

### 1.3 诊断金标准

病理组织学或细胞学检查结果、随访追踪结果。随访时间为3个月，超过随访时间未能确诊者不纳入统计分析。

### 1.4 剔除标准和禁忌症

严重的心肺功能不全、出血倾向、不合作者或知情不同意者。

### 1.5 获取病理诊断方法

经可曲支气管镜肺活检及细胞学检查、痰脱落细胞学检查、经皮肺穿刺活检、胸腔镜活检、开胸探查、手术切除等。

### 1.6 仪器

可曲支气管镜（BF-1T30、BF-1T240或BF- 1T260，日本，奥林巴斯）；腔内超声主机（ENDOECHO EU-M2000，日本，奥林巴斯）；超声探头驱动器（MAJ-935，日本，奥林巴斯）；径向支气管内超声探头（radial endobronchial ultrasound probe，UM-BS20-26R，外径2.0 mm；UM-DP20-25R，外径2.5 mm，日本，奥林巴斯）。

### 1.7 支气管内超声检查方法

术前准备同常规可曲支气管镜检查^[3]^。因周围型病变所连通支气管直径较小，可直接用探头进行探查。如探头贴壁不佳，出现空气干扰的伪像，可通过支气管镜活检通道注入普鲁卡因凝胶、生理盐水，或在探头外加上水囊（MH-246R，日本，奥林巴斯）。连接支气管内超声主机、探头驱动器和超声探头。驱动器旋转探头产生360o实时垂直图像。根据胸片或胸部CT初步确定病变部位，完成常规支气管镜检查后，将支气管镜送达预定位置，经活检通道送入超声探头，直至术者感觉有阻力，开始超声扫描，同时术者缓慢、匀速将探头往外拉出，观察超声图像。所得图像存于主机内，完成扫描后即可进行三维图像重建和显示。所有病理学结果由两位有经验的病理科医师做出诊断。

### 1.8 统计学处理

采用SPSS 13.0软件分析。计量资料采用Mean±SD表示，计数资料间的比较采用χ^2^检验，如四格表内期望频数＜1，或者＞1/5格子的理论频数＜5时，采用*Fisher*精确概率检验法。以*P*＜0.05为差异具有统计学意义。

## 2 结果

### 2.1 临床资料

入选的92例患者中，78例病灶良恶性诊断明确者纳入分析，男56例，女22例，年龄21岁-80岁，平均（58.01±13.20）岁，支气管内超声对肺部周围型病灶的总体检出率为84.8%（78/92）。病灶最后诊断及例数见表 1。

**1:** 78个肺周围型病灶的最后诊断和支气管充气征的关系 Relationship between diagnosis of peripheral lung lesions and air bronchogram in 78 patients

Final diagnosis	*n*	Air bronchogram		
		No	Regular	Irregular
Malignant lesions	47	22	1	24
Small cell carcinoma	3	2		1
Adenocarcinoma	5	2		3
Well differentiated adenocarcinoma	3	2		1
Moderately differentiated adenocarcinoma	10	3	1	6
Poorly differentiated adenocarcinoma	10	5		5
Papillary adenocarcinoma	1			1
Moderately differentiated squamous cell carcinoma	4	2		2
Poorly differentiated squamous cell carcinoma	1			1
Adenosquamous carcinoma	1	1		
Large cell carcinoma	1			1
Non-small cell cancer	5	3		2
Neuroendocrine carcinoma	1			1
Endometrial carcinoma	1	1		
Unclassified malignant lesions	1	1		
Benign lesions	31	3	25	3
Pneumonia	15		14	1
Pulmonary aspergillosis	2		2	
Bronchiectasis with infection	1		1	
Lung abscess	1		1	
Pulmonary tuberculosis	6	1	5	
Wegener's granulomatosis	1		1	
Chondromatous hamartoma of lung	1			1
Penicillium marneffei	1			1
Pulmonary fibrosis with infection	1		1	
Seborrheic pneumonia	1	1		
Inflammatory pseudotumor	1	1		
Total	78	25	26	27

恶性病变无支气管充气征者占46.8%（22/47），25例无支气管充气征的病灶中22例为恶性（占88%），其中非小细胞癌占43.9% (18/41)，低分化腺癌占50% (5/10)（图 1，图 2），4例中分化腺癌中2例无支气管充气征（图 3），小细胞肺癌占66.7%（2/3）（图 4），相应病理切片未见支气管充气相。恶性病变中不规则支气管充气征者占51.1%（24/47），以腺癌多见，占55.2%（16/29），病理切片见散在支气管充气相（图 5，图 6），类似征象亦见于2例中分化鳞癌和1例低分化鳞癌。恶性病变中无支气管充气征和不规则支气管充气征两者共计97.9%（46/47），仅1例恶性病变（中分化腺癌，图 7）表现为规则的支气管充气征（1.3%）。

**1:**
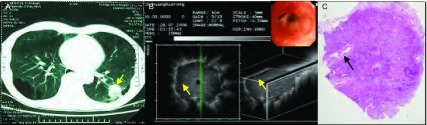
男，71岁，咳嗽胸痛1个月，低分化腺癌。A：CT见左肺下叶背段31 mm×22 mm圆形病灶；B：支气管镜下未见异常，支气管内超声于左下叶背段见病灶呈低回声，边界清晰，内部回声均匀，无支气管充气征，可见内部支气管狭窄（箭头所指）；C：病灶内可见支气管狭窄（箭头所指），瘤细胞大部分排列成实性巢状，分布密集，部分呈腺管样分化，灶性坏死，间质纤维增生，无支气管肺泡充气相（HE，×10）。 Male, 71 years old, cough and thoracic pain for 1 month, poorly differentiated adenocarcinoma. A: CT shows round lesion of 31 mm×22 mm at the apical segment of left lower lobe; B: No abnormality was found by bronchoscope, low echogenicity lesion was found at the apical segment of left lower lobe by endobronchial ultrasound probe, contour was clear, internal echo was homogeneous, no air bronchogram, bronchostenosis can be seen (at the arrow tip); C: Bronchostenosis can be seen in the lesion (at the arrow tip), tumor cells were mostly arranged as nestlike, intensively distributed, some of them showed crypt shaped differentiation, focal necrosis, and interstitial fibroplasias, no sign of air bronchogram (HE, ×10).

**2:**
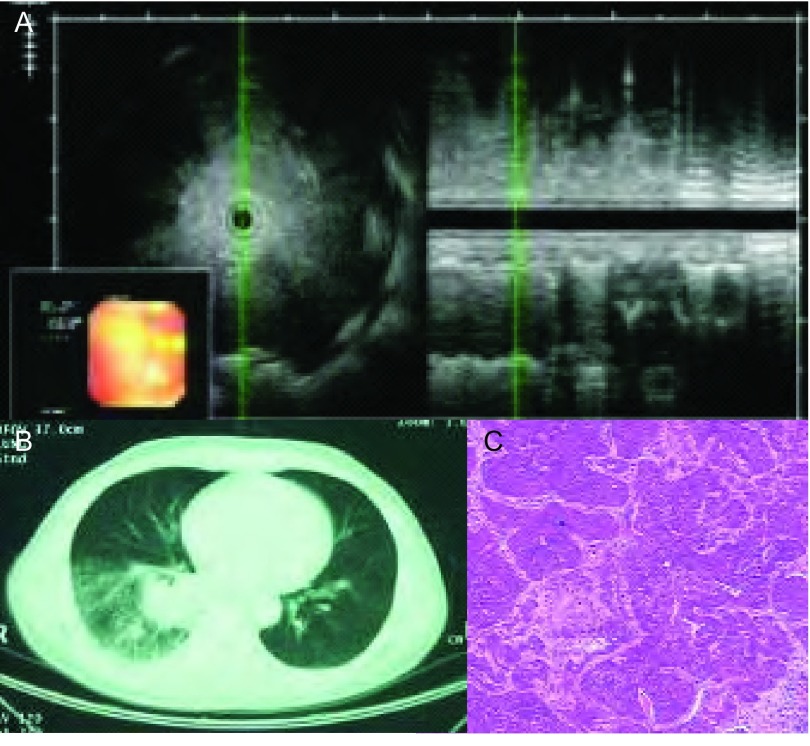
男，56岁，咳嗽，咳血丝痰3个月，低分化腺癌。A：腔内超声扫描见病灶内部回声均匀，无支气管充气征；B：CT见右肺下叶背段团块状高密度病灶；C：病理切片见肿瘤细胞分布密集排列成实性巢状，未见明显角化或腺腔形成伴间质纤维化，肿瘤组织内无充气相（HE，×40）。 Male, 56 years old, cough, and expectoration of blood tinged sputum for 3 months, poorly differentiated adenocarcinoma. A: homogeneous internal echo, no sign of air bron-chogram; B: CT shows a high-density lesion at the apical segment of right lower lobe; C: Tumor cells were mostly arranged as nestlike, intensively distributed by pathological section, no obvious cornification or formation of glandular cavity with interstitial fibrosis, no sign of inflation inside of tumor tissue (HE, ×40).

**3:**
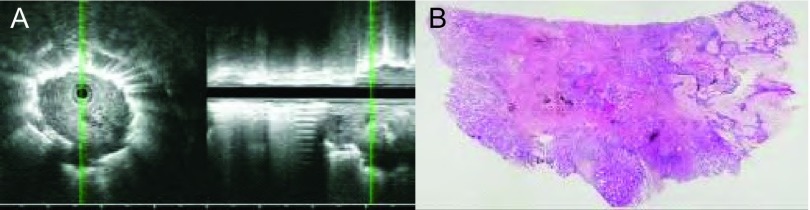
女，44岁，中分化腺癌。A：EBUS在右下肺前段探及低回声病灶，无支气管充气征；B：病理切片见成簇、成片密集排列的肿瘤细胞，无支气管充气相（HE，×10）。 Female, 44 years old, moderately differentiated adenocarcinoma. A: EBUS detected a low echo lesion at the front segment of right lower lobe, no sign of air bronchogram; B: Pathological section shows clustered high density tumor cell, no sign of air bronchogram (HE, ×10).

**4:**
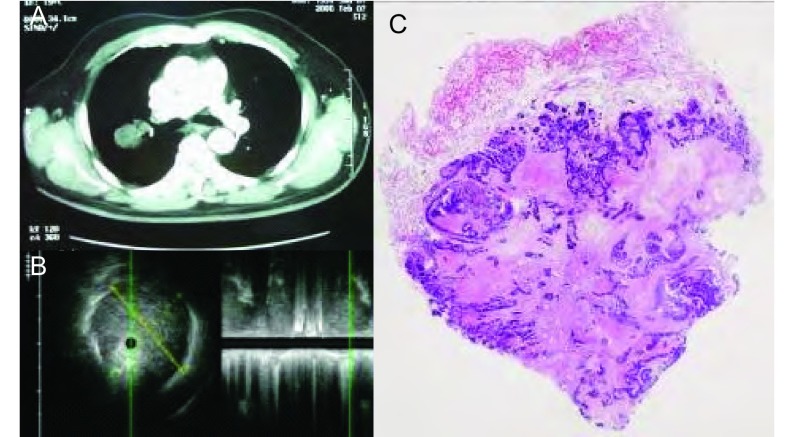
男，74岁，咳嗽咳痰1个月，小细胞肺癌。A：CT见右肺上叶圆形占位；B：支气管镜下未见异常，支气管内超声于右上叶前段见低回声病灶，内部无支气管充气征；C：病理切片可见肿瘤细胞紧密排列成片巢状，部分细胞挤压变形，灶性坏死，无支气管充气征（HE，×10）。 Male, 74 years old, cough and expectoration of sputum for 1 month, small cell lung cancer. A: CT shows a round occupation; B: No abnormality was found by bronchoscope, low echogenicity lesion was found at front segment of right upper lobe by endobronchial ultrasound, no air bronchogram was found; C: Tumor cells were mostly arranged as nestlike, intensively distributed by pathological section, some of them were crushed to distortion, focal necrosis, no sign of air bronchogram (HE, ×10).

**5:**
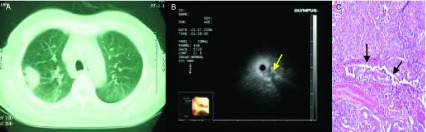
男，64岁，间断咳嗽咳痰1周，中分化腺癌。A：CT见右肺上叶后段36 mm×22 mm团块状高密度病灶；B：支气管镜下未见病灶，支气管内超声于右上叶后段c亚支见病灶内部回声不均匀，边界模糊中断，可见支气管充气征（黄色箭头）；C：病理切片见肿瘤细胞呈筛状、腺管样或小巢状，瘤巢间纤维增生伴炎细胞浸润，肿瘤内部支气管充气相（黑色箭头）（HE, ×40）。 Male, 64 years old, cough discontinuously for 1 week, moderately differentiated adenocarcinoma. A: CT shows a high density massive lesion of 36 mm×22 mm at apical segment of upper-right lobe; B: No lesion was found by bronchoscope, a lesion with internal heterogeneous echo was found in the c sub-segmental at back segment of right upper lobe by endobronchial ultrasound, border was fuzzy and interrupted, air bronchogram can be seen (at the yellow arrow tip); C: Pathological section shows tumor cells arranged in cribriform, crypt shaped or nestlike, fibroplasia among tumor nest complicated with inflammatory cell infiltration, air bronchogram found inside the tumor (at the black arrow tip)(HE, ×40).

**6:**
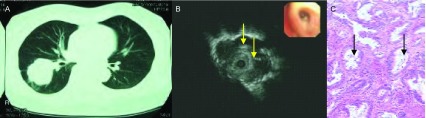
女，74岁，发现右肺占位1周，中分化腺癌。A：CT见右肺下叶背段占位圆形占位性病变；B：支气管镜下未见异常，支气管内超声见于右B6c亚段见低回声病灶，边界清晰，不规则，内部回声不均匀，支气管充气征分布不均（黄色箭头）；C：病理切片见纤维结缔组织内可见大量被覆异型上皮的、大小不等的腺管浸润性生长，伴灶性坏死，未见正常肺组织，肿瘤内部见支气管腔充气相（黑色箭头）（HE, ×40）。 Female, 74 years old, found occupation at the lung for 1 week, moderately differentiated adenocarcinoma. A: CT shows round occupying lesion at apical segment of right lower lobe; B: No abnormality was found by bronchoscope, low echo lesion was found at right B6c subsegment by endobronchial ultrasound, border was clear and irregular, internal echo was heterogeneous, air bronchogram was irregularly distributed (at the yellow arrow tip); C: Pathological section shows large quantity of allotype epithelium cover crypt infiltratively grow in different size complicated with focal necrosis, no normal lung tissue was found, air bronchogram was found inside the tumor (at the black arrow tip)(HE, ×40).

**7:**
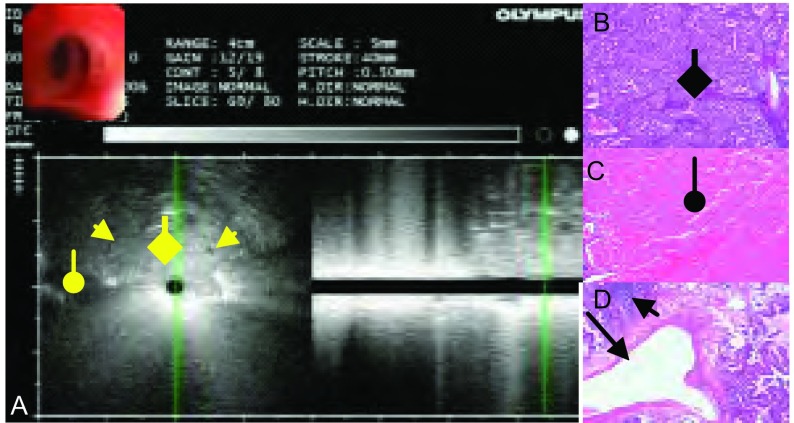
男，35岁，胸片示右下叶背段见60 mm×60 mm×40 mm团块状高密度病灶，中分化腺癌。A：边界不清，靠近探头的中央部分回声均匀，无支气管充气征（黄色菱形箭头），外周部分有同心圆状支气管充气征，肿瘤内部血管呈外压型狭窄或不规则形（黄色三角形箭头），外周回声不均，可见不规则低回声区（黄色圆形箭头），考虑为坏死；B：病理切片见肿瘤细胞分布密实均匀（黑色菱形箭头）；C：病理切片可见大片坏死灶（黑色圆形箭头）；D：病理切片见血管外压狭窄（长黑色三角形箭头），短黑色三角形箭头所指为肿瘤组织。 Males, 35 years old, chest film shows massive lesion in high density of 60 mm×60 mm×40 mm at the apical segment of right lower lobe, moderately differentiated adenocarcinoma. A: Border was fuzzy, central section near the detecting probe showed homogeneous echo, no air bronchogram (at the yellow rhombic arrow), concentric circles shaped air bronchogram was found at the peripheral part, blood vessels in the tumor show compressed striture or irregular shape (at the yellow triangle arrow), peripheral echo was heterogeneous, irregular hypoecho can be found (at the yellow circular arrow), necrosis was regarded; B: Pathological section shows tumor cells of high density evenly distributed (at the black rhombic arrow); C: Pathological section shows large area of necrotic lesion (at the black circular arrow); D: Pathological section shows blood vessels of compressed stricture (at the long black triangle arrow), the short black triangle arrow is pointing at the tumor tissue.

良性病变见规则支气管充气征者占80.6%（25/31）（图 8），无支气管充气征者或不规则支气管充气征各占3.8%（3/31），3例不规则支气管充气征的良性病变分别为肺炎、肺脓肿（图 9）、软骨瘤性错构瘤各1例。

**8:**
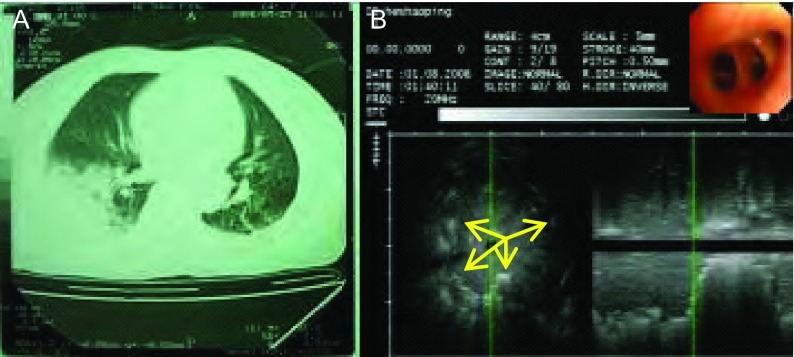
女，63岁，发热伴咳嗽咳痰1周，抗生素治疗1周后病灶明显吸收，肺炎。A：CT见右下肺实变；B：支气管镜下未见异常，右下叶外基底段行支气管内超声检查见病灶内部回声均匀，边界部分不清，部分呈锯齿状，支气管充气征位于周边，近似同心圆状规则分布（箭头）。 Female, 63 years old, fever complicated with cough and expectoration of sputum for 1 week, after 1 week of antibiotic therapy, the lesion was absorbed, diagnosed pneumonia. A: CT shows consolidation at the right lower lobe; B: No abnormality found by bronchoscope, endobronchial ultrasound in the lateral basal segment showed homogeneous echo in the lesion, the border was fuzzy, partially serrated, air bronchogram found at periphery part, distributed regularly at the shape similar to concentric circles (at the arrow tip).

**9:**
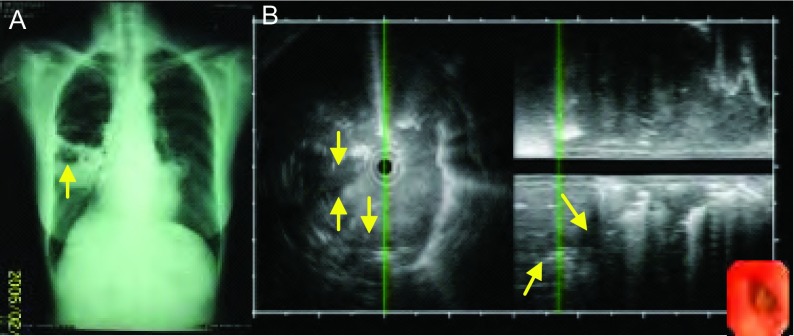
女，74岁，咳嗽、咳痰、发热10天，肺脓肿。A：胸片见右肺中叶和左肺舌叶片状阴影，内见空洞（箭头）；B：支气管镜下见右中叶外侧段粘膜充血，支气管内超声探查其远端肺实质见低回声病灶，边界欠清晰，内部回声不均匀，局部可见不规则更低回声区（箭头），考虑为坏死空腔。 Female, 74 years old, cough, and expectoration of sputum for 10 days, diagnosed pulmonary abscess. A: Chest film shows large shadow at the right middle lobe and left lingular lobe, cavity can be seen inside (at the arrow tip); B: Bronchoscope shows mucous hyperemia at the segment of right-middle lobe, endobronchial ultrasound at the distal lung parenchyma shows a low echo lesion with fuzzy border, heterogeneous echo, more lower echo area can be found in area (at the arrow tip), regarded as necrotic cavity.

### 2.2 不良反应

92例行支气管内超声检查的患者中，不良反应主要包括少量出血（25.22%），出血＜10 mL者8例，10 mL-50 mL者6例，经冰盐水、1:20 000肾上腺素、凝血酶等灌洗局部处理后出血均能停止；2例（2.17%）患者在用探头探查时出现胸痛，将探头退出即可缓解；4例（4.35%）患者出现咳嗽，加强局部麻醉后可缓解。

## 3 讨论

肺实变、肺不张、肺肿瘤均可导致肺气体减少或消失，密度增高，与周围正常充气肺组织的强反射比较，萎陷肺组织呈等回声或低回声，在少气肺或无气肺内进行超声探查，可清晰地观察到肺内组织的形态结构。病区内支气管仍通畅并含有气体时，支气管壁的界面反射、管腔内的空气反射以及周围充满液体的肺泡共同组成了斑点状或短线状的强回声，其后方多有多重反射（彗星尾征），为支气管充气征，而肺血管影无彗星尾征，可有搏动。当扫描对象比较大、表面平滑且垂直于声波束的传导方向、两种介质存在显著的声阻抗差时，这种现象更明显^[4]^。支气管充气征多由肺实质的病变导致，也有近端支气管阻塞，导致远端肺实质炎症与不张，其内支气管仍残留空气，形成支气管充气征，由于胸腔负压增加，可导致支气管扩张^[5]^。病区内支气管被分泌物填充时，在超声图像显示为支气管液相，无彗星尾征（图 1），当与肺动脉伴行时可形成“双管征”^[6, 7]^。

恶性肿瘤细胞成簇成片密集生长，挤占细支气管和肺泡空间，如果肿瘤内气体完全消失，病灶表现为低回声，与正常肝脾的回声接近，无支气管充气征。从表 2可见恶性病变46.8%（22/47）未见支气管充气征，25例无支气管充气征的病灶中22例为恶性（占88%），相应病理切片未见支气管充气相（图 1-图 4），Kurimoto^[8]^报道恶性肿瘤无支气管充气征者以低分化肿瘤和小细胞肺癌多见，但本研究中多种病理类型及各种分化程度均可见此表现（表 1）。Wang等^[9, 10]^报道快速生长的周围型小肺腺癌病灶内大多数无支气管充气征或小气泡征。提示这类肿瘤更需早期诊断和治疗干预。

**2:** 病灶性质与支气管充气征的关系 Relationship between diagnosis of lesions and air bronchogram

Final diagnosis	Air bronchogram			Total
	No(%)	Regular(%)	Irragular(%)	
Benign lesions	3 (3.8)	25 (32.1)	3 (3.8)	31
Malignant lesions	22 (28.2)	1 (1.3)	24 (30.8)	47
Total	25(32.0)	26 (33.4)	27 (34.6)	78

当病区内支气管被肿瘤包绕侵犯时，可导致管腔狭窄或闭塞^[11]^，如果细支气管内仍有残存的气体，EBUS下可见呈点线状高回声的支气管充气征，分布不规则，从病灶中央到外周均可见，以分化好的腺癌多见（图 5，图 6）。结合病理切片分析，点线状高回声主要为病灶内残余气体对超声波产生的强反射，与Kurimoto^[8]^报道符合，有报道^[12, 13]^结节内支气管气相为腺癌特征，本研究中其它不同大小、不同病例类型的肿瘤也可见支气管充气征，Gaeta^[14]^有类似报道。软骨、钙化灶也可表现为斑点状的高回声^[15]^，造成假象。

支气管内超声良性病变的线性离散支气管充气征的特征是在低回声的背景内，以探头所在支气管为轴心，规则、分层、呈同心圆状排列的短线状高回声影^[10, 15]^（图 8），基本保持细支气管和肺实质原来的结构形态，病区内的回声强度由内向外逐渐衰减^[11]^。肺炎病灶中央部分由于渗出液和细胞填充细支气管和肺泡空间，气体减少或消失，回声分布均匀，多无支气管充气征；边缘为正常充气肺实质与致密感染细胞层或纤维化交错区域，支气管充气征在病灶的外周分布多于中央部分，且近似同心圆状规则分布，与Chao等^[15]^的报道类似（图 8）。支气管内超声显示同心圆状的支气管充气征高度提示良性病变，偶尔在分化较好的腺癌中也可见到此征（图 7，中分化腺癌），需结合其它征象进行判断，该病灶仔细辨认可发现病灶内血管被推移、狭窄，而肺炎无此表现。肺脓肿内部回声和脓肿的坏死液化程度相关，早期以实性及不均匀低回声为主，当液化形成后可见不规则无回声区（图 9）。炎性假瘤、结核瘤边界较规整，内部回声强弱不均，后者有钙化灶时可见小点状、条状强回声伴有声影。

综上所述，支气管内超声图像于低回声病灶中无支气管充气征或出现不规则支气管充气征时，高度提示恶性病变，出现规则同心圆状分布的支气管充气征时，以良性病变可能性大。
